# Therapeutic Drug Monitoring and Pharmacogenetic Testing in Northern China

**DOI:** 10.3389/fphar.2021.754380

**Published:** 2021-11-02

**Authors:** Chunyan Zhang, Jing Lei, Yi Liu, Yu Wang, Lin Huang, Yufei Feng

**Affiliations:** ^1^ Department of Pharmacy, Peking University People’s Hospital, Beijing, China; ^2^ Peking University School of Pharmaceutical Sciences, Beijing, China

**Keywords:** therapeutic drug monitoring, pharmacogenetic testing, northern China, survey, public hospitals

## Abstract

**Background:** Therapeutic drug monitoring (TDM) and pharmacogenetic (PGx) testing are widely used as approaches to improve individualized (personalized) pharmacotherapy. Little is known about TDM and PGx testing services in China. This study is aimed to describe the TDM and PGx testing services in northern China, and to lay the foundation for improving these services.

**Methods:** We developed an electronic survey using online software and disseminated it to 32 public hospitals in northern China from May to July 2019. The data were analyzed using the Statistical Package for Social Sciences (SPSS) program (Ver.27.0).

**Results:** We collected 29 of the 32 questionnaires (90.6% response rate) from public hospitals in seven provinces of northern China. Twenty-two public hospitals (76%) utilized TDM; immune suppressants, antiepileptic drugs and anti-infective drugs were the main drugs monitored. The hospitals that did not provide TDM service were traditional Chinese medicine hospitals and hospitals with a smaller number of hospital beds. Seventeen public hospitals (58.6%) had PGx testing programs. The hospitals that did not offer PGx testing service had a smaller number of hospital beds and had fewer daily outpatients.

**Conclusion:** TDM is available in the vast majority of public hospitals in northern China, although mainly in tertiary hospitals. PGx testing, a newer approach, is less widely available. We recommend that more hospitals be encouraged to provide TDM and PGx testing services and more efforts be directed toward quality control, delivery of results and counseling of patients based on those results.

## Introduction

Personalized medicine is important for patient care for several reasons, including its ability to improve patient outcomes with respect to efficacy and safety (e.g., by reducing adverse reactions) and potentially reducing healthcare costs. Therapeutic drug monitoring (TDM) and Pharmacogenetic (PGx) testing are the two essential approaches that are commonly used for personalized medicine.

TDM involves the measurement of drug concentrations in biological samples in order to optimize and adjust drug dosage regimens. It is of particular utility with drugs that have a narrow therapeutic index or if a drug dosage does not reliably predict the serum concentration of the drug. It may also be helpful in settings where large variations in pharmacokinetic parameters occur among individuals or in individuals because of age, gender, environmental factors, genetic factors, and/or differences or changes in renal or hepatic function. TDM may be indicated when a new regiment is initiated, the dosage is changed, or serum drug concentrations are changing, perhaps because of changes in a patient’s clinical status or interacting medications are started or discontinued (Steven and Karel., 2016; Kang and Lee, 2009). TDM is also indicated if potential clinical or laboratory manifestations of toxicity occur or when adherence (compliance) is uncertain in patients receiving oral therapy.

PGx testing, the use of a patient’s genetic information to guide drug therapy, may subsequently decrease the patient’s risk for adverse drug effects and lack of drug effectiveness (FDA, 2008). Significant progress in PGx research has occurred over the past decade. PGx testing is recommended for many drugs before they are prescribed; such drugs include cetuximab, dasatinib, maraviroc, and trastuzumab (Zineh and Pacanowski, 2011).

In China, TDM has been recognized as being important for individualized treatment for more than 30 years. Some public hospitals began to use TDM during the 1980s, as did hospitals in Europe and the United States (Xiang-Lin, 2019). However, not all hospitals in China have used PGx testing. Nationwide surveys of TDM services have been reported for other countries, including Australia, the United Kingdom, Italy, Norway, Canada and Malaysia (Ab Rahman et al., 2013; Donna et al., 2018; Choi et al., 2019). Wei et al. documented the status of TDM of psychiatric drugs in China (Wei et al., 2013), but a nationwide survey on TDM and PGx testing in China has not been undertaken. We thus set out to survey the current use of TDM and PGx testing in public hospitals in northern China. Our goal was to identify the availability of TDM and PGx testing, and based on these findings, to propose measures for conducting TDM and PGx testing service more widely, in order to help optimize the allocation of medical resources in China.

## Methods

### Questionnaire

The survey was initiated by the National Health Commission and focused on public medical institutions. The questionnaire had 44 questions that included basic information, implementation of TDM and of PGx testing, and inquiry regarding testing of other drugs (the details are provided as supplementary data). The 9 basic information questions included the name and the nature of the unit, the average number of prescriptions per day, and the nature of the pharmaceutical service. The other 35 questions focused on TDM and PGx testing, Table 1 shows the condensed questionnaire. The survey took place from May to July 2019. The questionnaire was sent out electronically to the heads of pharmaceutical laboratory and clinical laboratory of 32 public hospitals in seven provinces and could be completed using WeChat or on a website.

**TABLE 1:** Items queried in the survey of TDM and PGx testing in Northern China hospitals.

Item number	Aspects	Focus of the item
1–9	Basic information	Name, province, rank of the hospital, number of hospital beds, total number of prescriptions per day, average daily outpatients, contents of pharmaceutical care, etc.
10–24	Implementation of TDM	General information regarding TDM (Drugs tested, number of years of TDM, information regarding TDM staff members, quality control, analytical methods, etc.).
		TDM Data (the top five drugs in 2016–2018, the total number of assays and abnormal value data). TDM counseling service, barriers for utilization of TDM.
25–43	Implementation of PGx testing	General information regarding PGx testing (drug targets tested, number of years of PGx testing, information regarding PGx testing staff members, quality control, analytical methods, etc.). PGx testing data (the top five drugs in 2016–2018, total numbers of tests). PGx testing counseling service, barriers for utilization of PGx testing, need for TDM and PGx testing
44	Other drug-related detection	Other drug-related testing programs that have been carried out

### Statistical Analyses

The data were analyzed using the Statistical Package for Social Sciences (SPSS) program (Ver.27.0). Descriptive statistics with frequencies, median and range were used when appropriate. Percentages were calculated based on the number of respondents for each question. The measurement data is expressed as the median and range. The Mann-Whitney U test was used because of skewed data and to evaluate differences in the availability of TDM and PGx testing services based on hospital characteristics. A *p* value less than 0.05 was considered statistically significant.

## Results

### Basic Information of the Responding Public Hospitals

**FIGURE 1:**
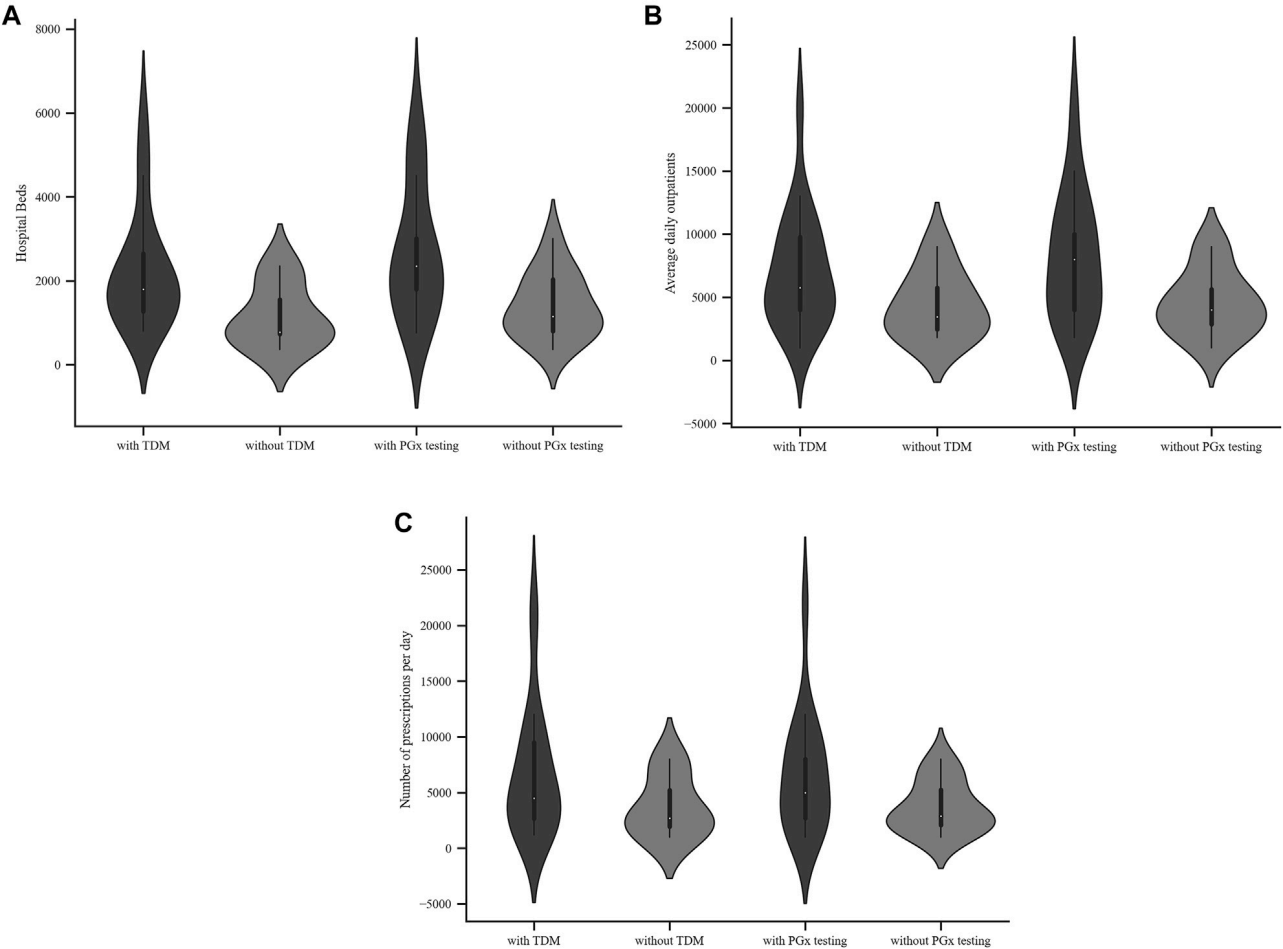
Characteristics of hospitals with or without TDM or PGx testing services **(A)** Hospital beds **(B)** Average daily outpatients **(C)** Total number of prescriptions per day.

Among the 29 respondents, 26 (90.6%) were tertiary A level hospitals and 3 (9.4%) tertiary B level hospitals. University affiliated hospitals accounted for 59.4%. The number of beds, the average daily outpatients and the total number of prescriptions per day in the hospitals are shown in Table 2 and Figure 1.

**TABLE 2:** Characteristics of Chinese hospitals responding to a survey of therapeutic drug monitoring.

Characteristic	Hospitals with TDM services no. (%)	Hospitals without TDM services no. (%)	*P* value^a^	Hospitals with PGx services no. (%)	Hospitals without PGx services no. (%)	*P* value^a^	
Province	*n* = 22	*n* = 7		*n* = 17	*n* = 12		
Beijing	12 (54.6)	5 (71.4)		11 (64.6)	6 (50.0)		
Hebei	3 (13.6)	0 (0.0)		1 (5.9)	2 (16.8)		
Liaoning	1 (4.6)	2 (28.6)		2 (11.8)	1 (8.3)		
Heilongjia	2 (9.0)	0 (0.0)		1 (5.9)	1 (8.3)		
Shandong	2 (9.0)	0 (0.0)		1 (5.9)	1 (8.3)		
Jilin	1 (4.6)	0 (0.0)		1 (5.9)	0 (0.0)		
Neimenggu	1 (4.6)	0 (0.0)		0 (0.0)	1 (8.3)		
University affiliation							
yes	13 (59.1)	4 (57.1)		13 (76.5)	4 (33.3)		
no	9 (40.9)	3 (42.9)		4 (23.5)	8 (66.7)		
Type of hospitals							
Level A Tertiary hospitals	20 (90.9)	6 (85.7)		15 (88.2)	11 (91.7)		
Level B Tertiary hospitals	2 (9.1)	1 (14.3)		2 (11.8)	1 (8.3)		
Hospital Beds Median (Range)	1800 (800–6,000)	776 (365–2,350)	***0.02***	2,350 (760–6,000)	1,150 (365–3,000)	***0.02***	
Average daily outpatients Median (Range)	2,750 (1,000–20,000)	3,437 (1800–9,000)	*0.12*	8,000 (1800–20,000)	5,000 (1,000–22,000)	***0.04***	
Total number of prescriptions per day Median (Range)	4,500 (1,200–22,000)	2,704 (1,000–8,000)	*0.18*	4,000 (1,000–9,000)	2,893 (1,000–8,000)	*0.10*	

n: indicates the number of respondents; a: Mann–Whitney U test; bold-italic data: p<0.05; italic data: P≥0.05.

### Status of Therapeutic Drug Monitoring Service

Among the 29 public hospitals, 22 (75.9%) provided a TDM service. The hospitals that provided TDM service had significantly more beds (Mann-Whitney U test, *p* < 0.05), higher average daily outpatients and higher number of prescriptions per day, though there was no statistical difference (Mann-Whitney U test, *p* ≥ 0.05), as shown in Table 2. The respondents that did not perform TDM service were specialized hospitals, including traditional Chinese hospitals and hospitals that specialized in oncology.

The drugs monitored in each hospital ranged from less than five to more than twenty, and the time period of TDM was from less than 5 years to 40 years. The number of testing technologists (predominantly with undergraduate and master’s degree) was generally less than five. In 22 laboratories the analytical methods involved liquid chromatography and immunoassay, and immunoassay was more common. Among the hospitals, 81.8% carried out Quality Control (QC) and were mainly at the national level, as shown in Table 3.

**TABLE 3:** Current status of TDM in hospitals that responded to the survey.

Characteristic	No. (%) of respondents (22)
Number of drugs monitored	
< 5	7 (31.8)
5–9	10 (45.5)
10–14	1 (4.5)
15–19	2 (9.0)
>20	2 (9.0)
Number of years	
< 10	6 (27.3)
10–20	6 (27.3)
20–30	6 (27.3)
30–40	4 (18.1)
Number of test members	
1–2	7 (31.8)
3–5	11 (50.0)
6–10	3 (13.6)
>10	1 (4.5)
Analytical method/instruments	
HPLC/MS	10 (45.5)
i1000 or i2000	8 (36.4)
HPLC	7 (31.8)
Viva-E	5 (22.7)
TDX	2 (9.0)
IMX	1 (4.5)
Other	8 (36.4)
Quality control	
No	4 (18.2)
Yes	18 (81.8)
At the national level	15 (83.3)
At the provincial level	2 (11.1)
Others	1 (5.6)
TDM clinic	
Yes	2 (9.1)
No	20 (90.9)
Clinical needs	
Very much needed	11 (37.9)
Yes	18 (62.1)
No	0 (0)

HPLC/MS: High performance liquid chromatography-mass spectrometry; i1000 or i2000: Automatic chemiluminesce analyzer(i1000 or i2000); HPLC: High performance liquid chromatography; Viva-E: Automatic immune analyzer (Viva-E); TDX: Fluorescence polarization immunoassay analyzer (TDX); IMX: Microparticle enzyme immunoassay (IMX analyzer).

**FIGURE 2:**
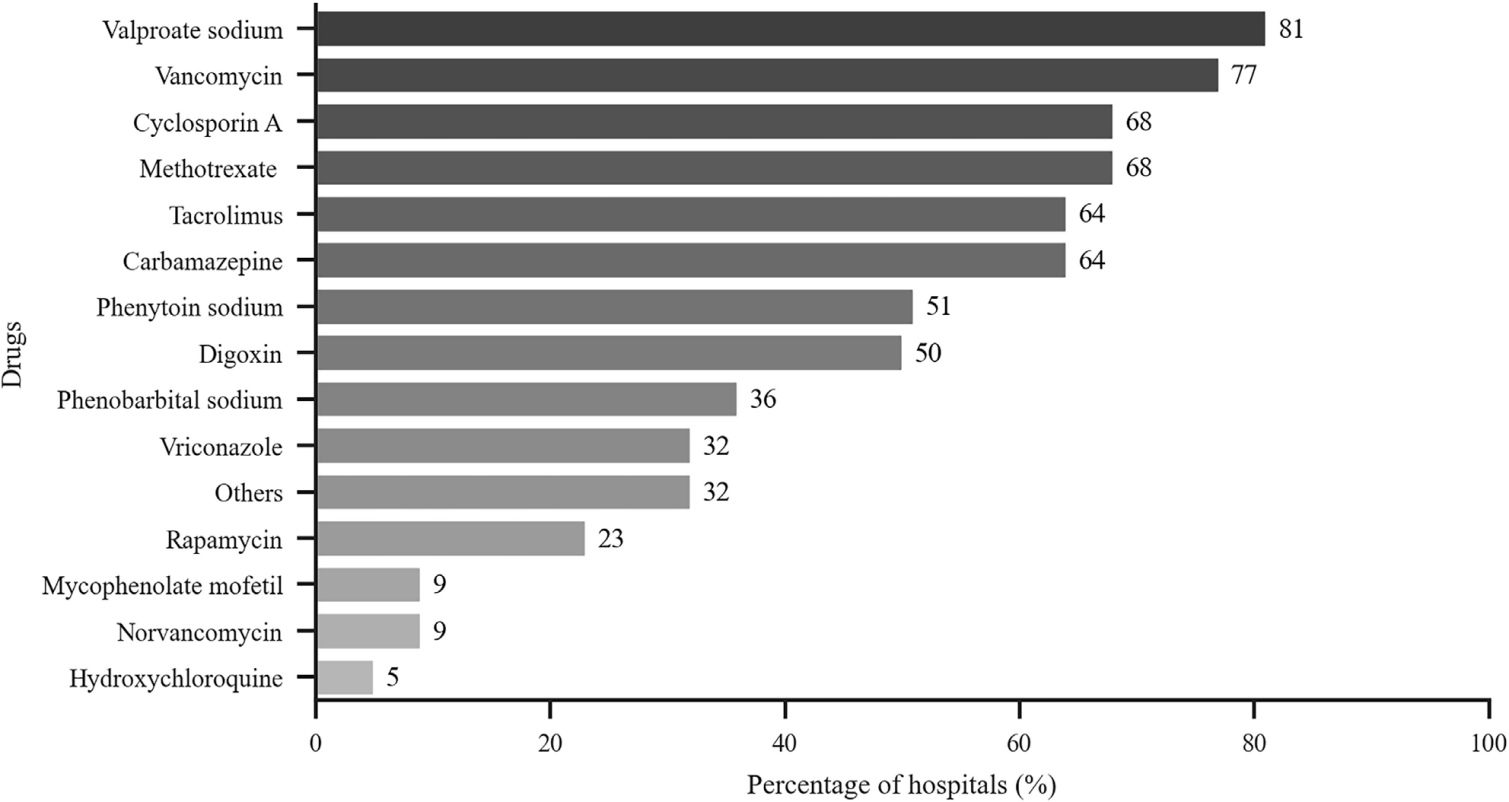
Drugs monitored by hospitals that perform TDM. *x*-axis: % of hospitals with TDM services that perform test related to the indicated drugs (*n* = 22).

As shown in Figure 2, the monitored drugs primarily included cyclosporine A, tacrolimus, mycophenolate mofetil, rapamycin, vancomycin, norvancomycin, voriconazole, methotrexate, hydroxychloroquine, carbamazepine, valproate sodium, phenobarbital, phenytoin sodium, and digoxin. Valproate sodium, vancomycin, cyclosporine A, tacrolimus and methotrexate were the most frequently monitored drugs from 2016 to 2018. Those five drugs were monitored in more than half of these hospitals. As shown in Table 4, the total number of assays increased during those years.

**TABLE 4:** The number of people tested for the top five drugs monitored in 2016–2018.

Drug monitored	2016	2017	2018	Total
Cyclosporin A	61,941	67,344	105,333	234,618
Tacrolimus	21,593	28,221	39,690	89,504
Methotrexate	10,773	13,423	15,531	39,727
Valproate sodium	8,003	8,734	9,259	25,996
Vancomycin	6,318	6,803	8,012	21,133

**FIGURE 3:**
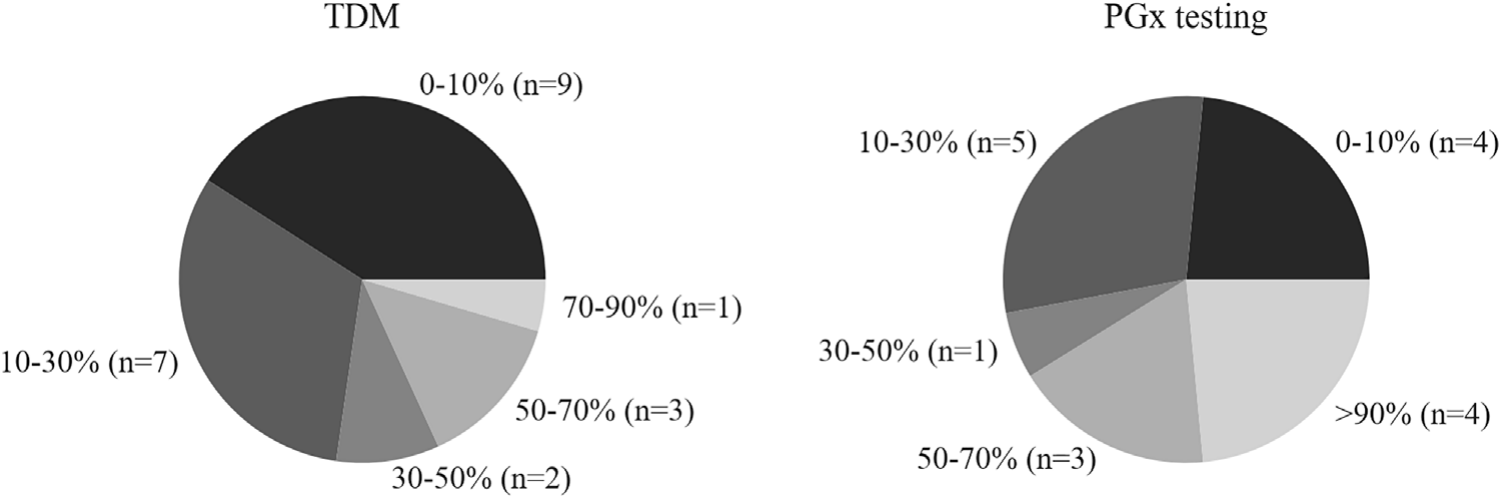
Clinical intervention rate (the ratio of intervention number to total TDM or PGx) of hospitals with TDM and PGx testing.

The proportion of abnormal value in 2016–2018 accounted for 1.32, 1.30, and 1.68%, respectively. A TDM service should include not only assays but also counseling as interpretation of the results is important for clinical decision making. The survey revealed that clinical pharmacists interpreted the results in 18 hospitals (81.8%), but as shown in Figure 3, the clinical intervention rate (the proportion of intervention quantity to total amount of TDM) was less than 30% in 16 institutions.

### Status of Pharmacogenetic Testing

Of the 29 public hospitals, 17 (58.6%) provided a PGx testing service. Comparison of hospitals that did or did not provide a PGx testing service revealed a similar trend to what was observed for TDM service. Hospitals that provided a PGx testing service had more beds and daily outpatients (Mann-Whitney U test, *p* < 0.05). Those hospitals had more prescriptions per day but this finding was not statistically significant (Mann-Whitney U test, *p* ≥ 0.05), as shown in Table 2 and Figure 1.

Less than ten drug-related genes were tested in 8 (52.9%) hospitals. PGx testing was conducted for less than 10 years in 16 (94.1%) of the hospitals. The number of testing technologists was less than five in 14 (82.4%) of the hospitals. The testing technologists had mainly master’s and doctoral degree, and most of the technologists had a senior professional title. The reported laboratory techniques included *in situ* hybridization, PCR-direct sequencing, PCR-pyrophosphate sequencing, PCR-fluorescence quantitation and PCR-gene chip method. Table 5 shows that 15 (88.2%) hospitals carried out QC. The QC was mainly at the national level.

**TABLE 5:** Current status of PGx testing in hospitals that responded to the survey.

Characteristic	No.(%)of respondents (17)
Number of items monitored	
< 5	5 (29.4)
5–9	4 (23.5)
10–14	2 (11.8)
15–19	2 (11.8)
>20	4 (23.5)
Number of years	
< 5	9 (52.9)
5–10	7 (41.2)
11–20	1 (5.9)
>20	0 (0)
Number of stafft members in PGX testing laboratories	
1–2	8 (47.1)
3–5	6 (35.3)
6–10	3 (17.6)
>10	0 (0)
Analytical method	
*In situ* hybridization method	9 (52.9)
PCR- fluorescence quantitative method	6 (35.2)
PCR- gene chip method	5 (29.4)
PCR- pyrophosphate sequencing method	4 (23.5)
PCR-direct sequencing method	1 (5.9)
Quality control	
No	2(11.8) 15(88.2)
Yes	
At the national level	14 (93.3)
At the provincial level	1 (6.7)
Clinical needs	
Very much needed	4 (13.8)
Yes	22 (75.9)
No	3 (10.3)

**FIGURE 4:**
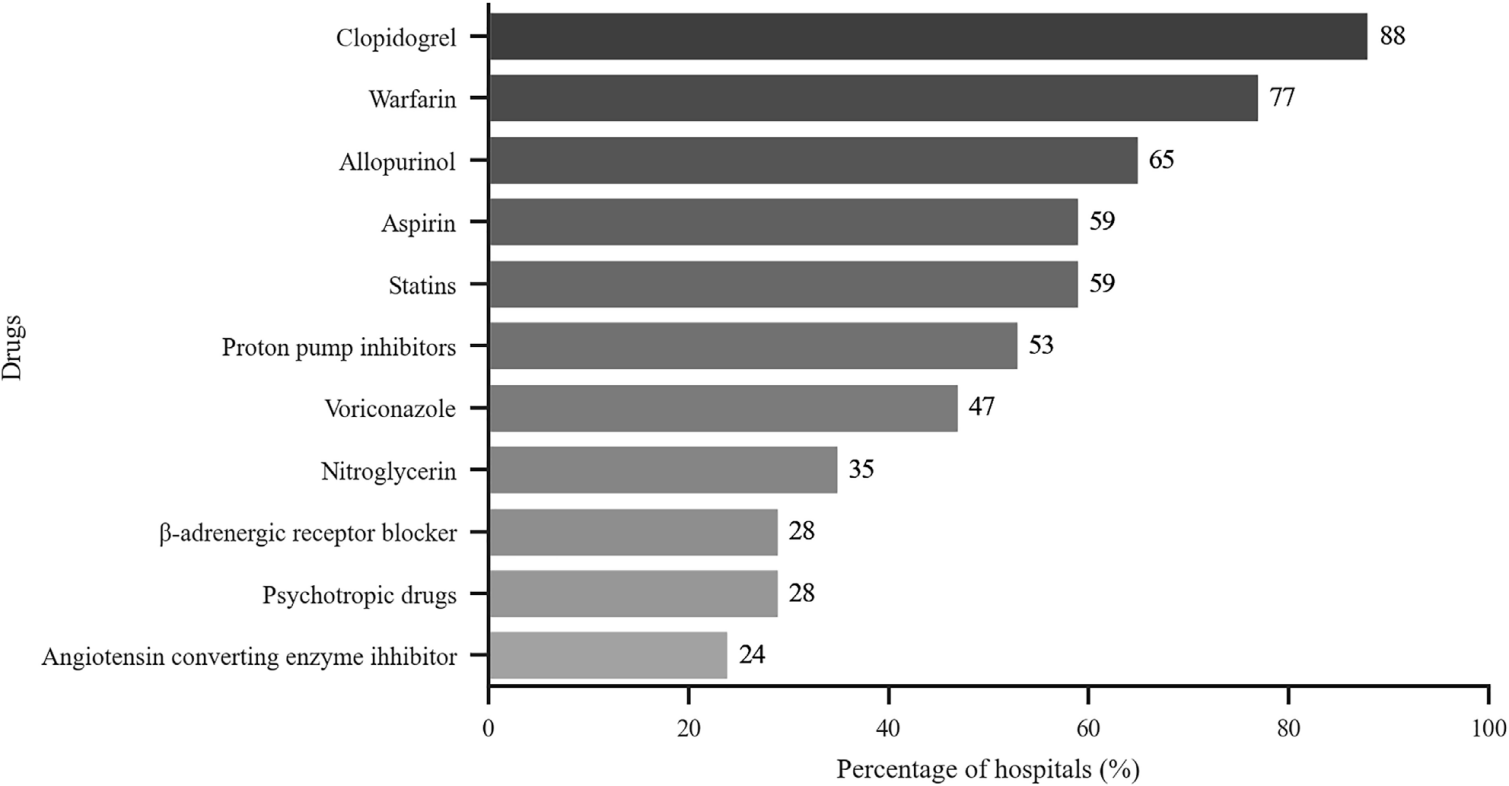
Drugs tested by hospitals that perform PGx testing. *x*-axis: % of hospitals with PGx services that perform test related to the indicated drugs (*n* = 17).

The drugs evaluated by PGx testing included allopurinol, antihypertensive drugs, aspirin, clopidogrel, nitroglycerin, proton pump inhibitors, psychotropic agents, statins and warfarin. As shown in Figure 4, the focus of more than half of the hospitals was on allopurinol, aspirin, clopidogrel, proton pump inhibitors, statins and warfarin. Table 6 shows that clopidogrel, folic acid, nitroglycerin, statins and warfarin were the top five drugs that were monitored from 2016 to 2018.

**TABLE 6:** The number of the top five drugs evaluated in PGx testing in 2016–2018.

Drug tested	Number of people tested			Number of assays		
	2016	2017	2018	2016	2017	2018
Clopidogrel	16,604	20,203	23,609	36,525	43,924	50,605
Warfarin	7,876	9,514	10,530	22,449	26,702	28,939
Folic acid	5,539	7,042	7,736	5,871	7,604	8,088
Nitroglycerin	3,695	3,736	3,804	3,695	3,736	3,804
Statins	2,651	3,309	4,429	4,766	7,057	9,742

With respect to counseling for PGx testing, the survey indicated that counselor support was available from pharmacists in 16 hospitals (94.1%), but as shown in Figure 3, the clinical intervention rate (the proportion of intervention quantity to total PGx testing amount) was less than 30% in 9 (53%) of the hospitals.

### Barriers to Therapeutic Drug Monitoring and Pharmacogenetic Testing

**FIGURE 5:**
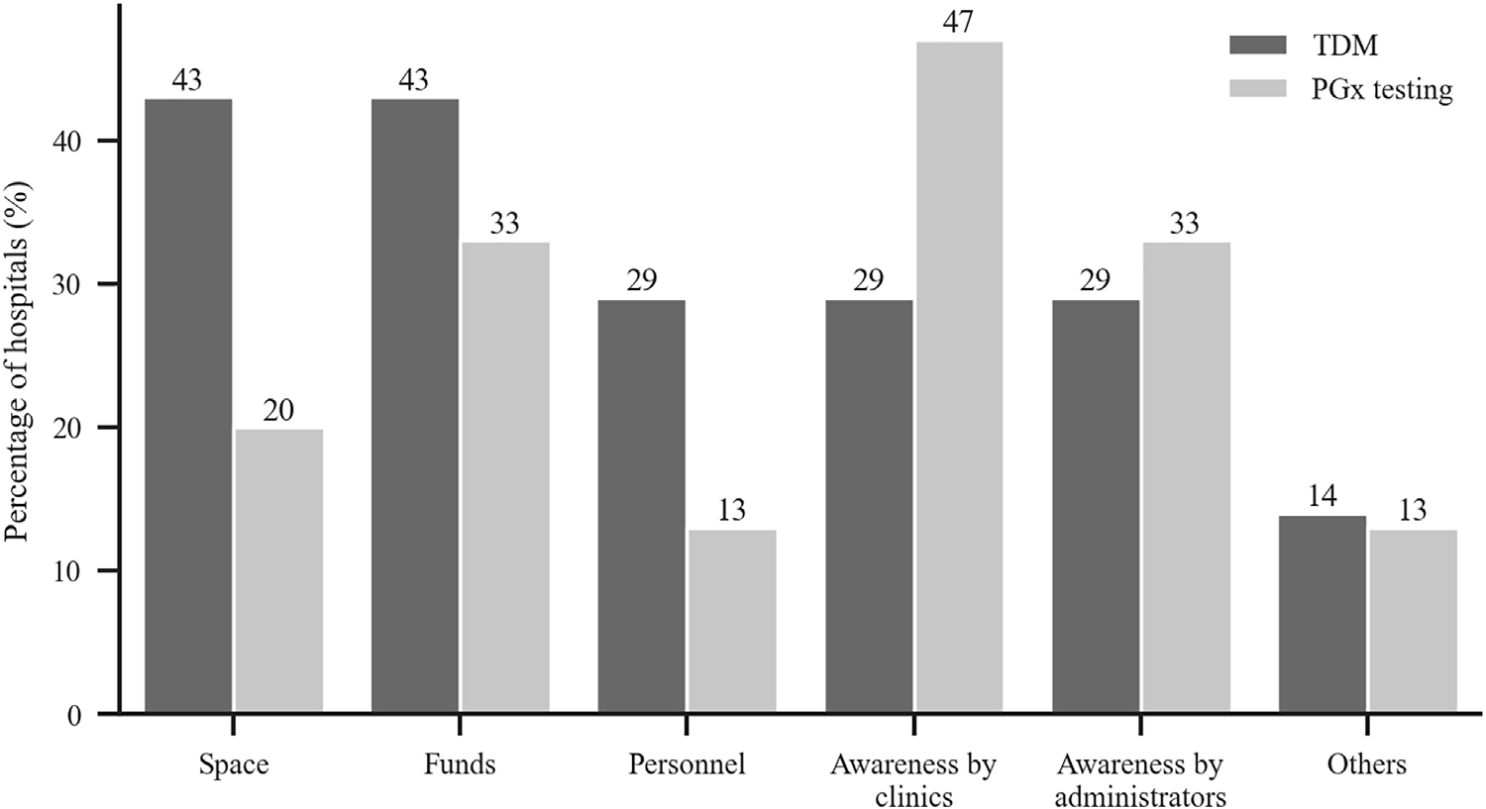
Reported reasons for not conducting TDM and PGx testing were lack of each of the following category. *y*-axis: % of hospitals without TMD or PGx service (TDM: *n* = 7, PGx: *n* = 12).

Figure 5 shows that the barriers for not carrying out TDM and PGx testing services included lack of funds, lack of pharmaceutical personnel, lack of awareness by clinics, and lack of awareness by administrators and others.

## Discussion

This study provides new information regarding the current status of TDM and PGx testing service in northern China. We found that TDM service was widely available in tertiary public hospitals in northern China, a result consistent with data from other countries (Norris et al., 2010; Ab Rahman et al., 2013; Donna et al., 2018; Choi et al., 2019). The hospitals not providing TDM service were traditional Chinese medicine hospitals or ones with a smaller number of hospital beds.

PGx testing was developed in China in recent years for individualized drug adjustment. This survey revealed that PGx testing was performed only in some tertiary A level hospitals. The hospitals not providing PGx service had fewer hospital beds and less average daily outpatients. In addition, the testing personnel for PGx testing generally had more education and higher professional titles, which was related to the high requirements of gene detection technology. Thus, in China, PGx testing is still in the early stage but its use will likely increase, as reflected in the increasing usage of PGx between 2016 and 2018 (Table 6).

The survey showed that TDM was not widely available for many drugs in certain hospitals (with less than 10 reported in about 80% hospitals). However, those drugs were commonly monitored, e.g., eight frequently monitored drugs were monitored by ≥ 50% of all hospitals. The most frequent drugs tested were immunosuppressants, antibiotics, antiepileptic drugs and cardiovascular drugs, which were similar to other reports (Ab Rahman et al., 2013; Donna et al., 2018; Choi et al., 2019). The 5 most frequently drugs from 2016 to 2018 were cyclosporin A, tacrolimus, methotrexate, valproate sodium and vancomycin. As other studies have reported, TDM was more widely used in patients with chronic disease and/or infectious diseases (Ab Rahman et al., 2013; Wei et al., 2013; Donna et al., 2018; Choi et al., 2019). We found that PGx testing was widely used to assess clopidogrel, warfarin and statins (>50% of all responders); these drugs were among those in the top five drugs assessed by PGx. Clopidogrel and warfarin were the most commonly used anticoagulants. Since gene mutations that affect the efficacy of those two drugs is higher in Chinese people, PGx testing is considered essential for individualized drug therapy (Mohamed and Julie, 2013; Justin et al., 2017; Zhang et al., 2017). Statins were often prescribed lipid-lowering drugs, especially in elderly patients. PGx detection can be helpful for selection and dosing of statins, in order to increase efficacy and reduce adverse reactions (Kitzmiller et al., 2016).

The accuracy of results was crucial for clinical adjustment of individualized treatment and QC was indispensable to guarantee the accuracy of the results. To ensure credibility of procedures (Rogers et al., 2010), most hospitals participated in evaluation of inter-laboratory QC in addition to internal QC. The survey revealed that 84 and 88% of the hospitals utilized inter-laboratory QC for TDM and PGx testing, respectively. More than 80% of the hospitals carried out QC at the national level, and of which over 90% were nationally accredited. Other studies have obtained data regarding QC. For example, Rahman et al. reported that most hospitals in Malaysia participated in quality assurance (QA) programs but these programs varied among the hospitals. One-third of the hospitals with QA programs participated in international QA programs while the rest conducted this locally (Ab Rahman et al., 2013). Rihwa et al. reported that most laboratories surveyed in South Korea conducted internal QC using commercialized QC with 20–29% of respondents not participating in external QA (Choi et al., 2019). We suggest that all laboratories participate in the national inter-laboratory QC system. And at the same time, the pass rate of QC should be assigned as the assessment index of the laboratory.

Counseling is the core of follow-up of results from TDM and PGx testing. This survey found that results of TDM and PGx testing were explained by clinical pharmacists in more than 80% of the hospitals. The abnormal value of TDM was the focus. Clinical pharmacists learned the reasons for abnormal results through understanding the patient’s disease status, details of drug usage and lifestyle changes, and subsequently offered suggestions for adjustment of the drugs. Other studies have mentioned counseling services. Rihwa et al. employed software in a TDM counseling service in South Korean (Choi et al., 2019) and noted racial differences in pharmacokinetics and pharmacodynamics. Future studies to obtain pharmacokinetic and pharmacodynamic information from Chinese patients by increased utilization of TDM are needed to improve health care in China. For PGx testing, the aim was to predict the efficacy and adverse reactions of drugs before administration. Pharmacists interpreted the results and developed a treatment plan with the physicians. In order to improve the professionalism of the result interpretation, on the one hand, the pharmacists should work closely with clinical laboratory department, and on the other hand, professional learning and training for the pharmacists should be strengthened.

The major barriers to TDM that were reported in our survey were inadequate space and insufficient funds, lack of pharmaceutical personnel and clinical awareness. For PGx testing, the main blocks were lack of clinical awareness and funds. The most important reason for hospitals not to have TDM and PGx testing services was the absence of laboratories. When the survey asked the clinical needs for TDM and PGx testing services, all the responding hospitals gave positive answers for a TDM service and 90% noted clinical needs for a PGx testing service. Rahman et al. reported in their survey in Malaysia that 56% of hospitals provided TDM service but that the drug concentration measurements were done in other hospitals (Ab Rahman et al., 2013).

We conclude that based on clinical needs, two approaches might be considered to more widely implement TDM and a PGx testing service. Firstly, regional pharmaceutical service centers could be created with the responsibility for assaying samples from hospitals in the surrounding area. Secondly, larger hospitals with laboratories could receive samples from hospitals that lack laboratories.

This study used a survey to investigate TDM and PGx testing services in 32 public hospitals in seven provinces in northern China. The relatively small sample size (in part, because of the large amount of data required in each questionnaire) is a limitation of the study but the data provide new, and we believe useful, findings regarding the current status of such services in this region in China.

## Conclusion

This survey showed that the majority of the tertiary public hospitals in northern China provide TDM service, but most hospitals monitored less than 10 drugs with the choice of drugs appropriate for their specific clinical requirements. PGx testing was only recently provided in northern China, and was not available in many hospitals. Based on the clinical needs reported in this study, more hospitals should be encouraged to provide TDM and PGx testing services and more drugs should be included. Perhaps establishment of independent testing centers separate from individual hospitals should be considered. In the meantime, the existing laboratories require more effort directed at quality control and counseling services to help interpret results and improve patient care.

## Data Availability

The original contributions presented in the study are included in the article/Supplementary Material, further inquiries can be directed to the corresponding author.
