# Supplementary material for: Therapeutic Drug Monitoring and Pharmacogenetic Testing in Northern China
Source: Front Pharmacol. 2021 Nov 2;12:754380. doi: 10.3389/fphar.2021.754380 (PMC8593476; doi:10.3389/fphar.2021.754380)
Supplement: Supplementary file 1 [file DataSheet1.DOCX]

***Questionnaire of pharmaceutical-related testing services***

***in the public hospitals***

**Instructions:** The survey is initiated by the National Health Commission and aimed to describe the TDM and PGx testing services in the public hospitals in northern China. The questionnaire consists of four parts: basic information, TDM service, PGx testing service, and other drug-related testing services. The survey does not involve personal information. It can be completed within 10minutes.

**I. Basic information**

1. Hospital Name

Province

Beijing, Hebei, Liaoning, Heilongjiang, Shandong Jilin, Neimenggu

1. University affiliation

Yes No

1. Type of hospitals

Level A Tertiary hospital, Level B Tertiary hospital, Secondary hospital,

Community hospital, Others

1. Pharmaceutical jobs

Drug dispensing, Traditional Chinese medicine (TCM) tisanes, Clinical pharmacy,

Pharmacy administration, Pharmaceutical preparation, Pharmaceutical laboratory,

PIVAs, Others

1. Hospital beds
2. Average daily outpatients
3. Total number of prescriptions per day
4. The number of pharmacists accounts for 8% of total health professionals

Yes No

1. Content of pharmaceutical care

Outpatient drug consultation, Medication guidance for discharged patients, Pre-prescription check, Prescription comment, Medicine clinic, Internet medication guidance, Lectures on medicine, Clinical pharmacists for personalized therapy, Clinical medication monitoring and evaluation, Monitoring and evaluation of antibiotic use, Evaluation of surgical prophylactic drugs, Collection and reporting of adverse reactions, TDM and PGx testing services

**II. Status of TDM service**

1. TDM service

Yes No

1. Drugs monitored of TDM

Cyclosporine A, Tacrolimus, Mycophenolate mofetil, Rapamycin, Vancomycin, Norvancomycin, Voriconazole, Methotrexate, Hydroxychloroquine, Carbamazepine, Valproate sodium, Phenobarbital, Phenytoin sodium, Digoxin, Others

1. Time of TDM

<5 years, 5-10 years, 10-15 years,15-20 years, 20-25 years, 25-30 years, 30-35 years, 35-40 years, >40 years

1. Number of testing technologists

1-2, 3-5, 5-10, >10

1. Educational background and professional title of testing technologists

Doctor Master Undergraduate and below

Senior title Medium-grade title Primary title

1. Inter-laboratory quality control

Yes No

Level of QC

National level, Province level, Others

Percent of pass

100%, 90-99%, 80-89%, 60-79%, <60%

1. Number of drugs monitored of TDM

<5, 5-9, 10-14, 15-19, >20

1. The number of the first five drugs monitored of TDM in the last three years (based on 2018)

Drugs 2106 2017 2018

1

2

3

4

5

1. The total number of TDM and abnormal value in recent three years

2016 2017 2018

The total number of TDM

The number of abnormal value

1. Instruments of TDM

i2000, i1000, IMX, TDX, HPLC, GC, HPLC/MS, GC/MS, Others

1. Clinical pharmacists interpreted the results of TDM

Yes No

1. Clinical intervention rate (the proportion of intervention quantity to total amount of TDM)

<10%, 10-30%, 30-50%, 50-70% , 70-90%, >90%

1. The departments deliver samples for TDM

1. Barriers to TDM

Lack of spaces, Lack of funds, Lack of pharmaceutical personnel, Lack of awareness by clinics, Lack of awareness by administrators, Others

1. Clinic needs for TDM

No, Yes, Very much needed

**III. Status of PGx testing service**

1. PGx testing service

Yes No

1. Drugs tested of PGx

Allopurinol, Aspirin, Angiotensin Converting Enzyme inhibitor, β-adrenergic receptor blocker, Clopidogrel, Nitroglycerin, Proton pump inhibitors, Psychotropic agents, Statins, Voriconazole, Warfarin, Others

1. Time of PGx testing

<5 years, 5-10 years, 10-15 years, 15-20 years, >20years

1. Number of testing technologists

1-2, 3-5, 5-10, >10

1. Educational background and professional title of testing technologists

Doctor Master Undergraduate and below

Senior title Medium-grade title Primary title

1. Inter-laboratory quality control

Yes No

Level of QC

National level, Province level, Others

Percent of pass

100%, 90-99%, 80-89% , 60-79% , <60%

1. Number of drugs monitored of PGx testing

<5, 5-9, 10-14, 15-19, >20

1. The number of the first five drugs monitored of PGx testing in the last three years (based on 2018)

Drugs and number of people tested

Drugs 2106 2017 2018

1

2

3

4

5

Drugs and number of assays

Drugs 2106 2017 2018

1

2

3

4

5

1. The total number of PGx testing in recent three years

2016 2017 2018

The total number of people tested

The total number of assays

1. Techniques of PGx testing

PCR-direct sequencing, PCR-pyrophosphate sequencing, PCR-fluorescence quantitation, PCR-gene chip method, PCR-electrophoretic analysis, PCR-high resolution dissolution curve method, PCR- allelic specificity method, PCR-in situ hybridization, Others

1. Clinical pharmacists interpret the results of PGx testing

Yes No

1. Clinical intervention rate (the proportion of intervention quantity to total amount of PGx testing)

<10%, 10-30%, 30-50%, 50-70%, 70-90%, >90%

1. The departments deliver samples for PGx testing

1. Barriers to PGx testing

Lack of spaces, Lack of funds, Lack of pharmaceutical personnel, Lack of awareness by clinics, Lack of awareness by administrators, Others

1. Clinic for TDM and PGx testing

Yes No

1. The frequency of clinic for TDM and PGx testing

Once a week, Twice a week, Three times a week, Four times a week, Five times a week

1. Drugs needed for TDM in community hospitals

Immunosuppressants, Antimicrobial agents, Antiepileptic drugs, Cardiovascular drugs, Antiasthmatic drugs, Antifungal drugs, Antitumor drugs, Antituberculous drugs, Others

1. Drugs needed for PGx testing in community hospitals

Antitumor drugs, Cardiovascular drugs, Digestive drugs, Endocrine drugs,

Rheumatic immune drugs, Antimicrobial agents, Narcotic analgesic drugs,

Psychotropic drugs, Physical examination items (alcohol metabolism, etc.),

Others

43. Clinic needs for PGx testing

No, Yes, Very much needed

**IV. Other testing items**

1. Other drug-related testing items besides TDM and PGx testing
